# Loss of expression and function of Gβγ by GNB1 encephalopathy-associated L95P mutation of the Gβ_1_ subunit

**DOI:** 10.3389/fphar.2025.1592012

**Published:** 2025-05-09

**Authors:** Haritha P. Reddy, Tal Keren-Raifman, Galit Tabak, Nathan Dascal, Daniel Yakubovich

**Affiliations:** ^1^ School of Medicine, Tel-Aviv University, Tel Aviv, Israel; ^2^ Sagol School of Neuroscience, Tel-Aviv University, Tel Aviv, Israel; ^3^ The Adelson School of Medicine, Ariel University, Ariel, Israel; ^4^ Neonatology Department, Laniado Hospital, Netanya, Israel

**Keywords:** GIRK channels, GNB1, mutation, modeling, docking

## Abstract

**Background:**

G-proteins areindispensable regulators of cellular signaling, with G-protein-gated inwardly rectifying potassium channels (GIRK) as key effectors. *GNB1* encephalopathy (GNB1E) is a congenital neurological syndrome resulting from mutations in the GNB1 gene, encoding the Gβ_1_ subunit of G-proteins trimer (Gαβγ). GNB1E manifests as a global developmental delay, accompanied by tonus disturbances, ataxia, and epilepsy.

**Methods:**

We utilized the *Xenopus laevis* oocyte heterologous expression system to investigate the impact of the L95P mutation in Gβ_1_ (Gβ_1_-L95P) on the activation of neuronal GIRK channels GIRK2 and GIRK1/2. Mutant and wild-type (WT) Gβ_1_ RNAs were co-injected with RNAs encoding the Gγ_2_ and GIRK channel subunits. The expression levels of both Gβ_1_ and the channel proteins, as well as the channel activity, were systematically monitored. Additionally, rigid-body docking was used to model the GIRK1/2–Gβγ complex, evaluating L95P’s effect on channel–Gβγ interaction, Gβγ stability, and Gβγ–effector affinity.

**Results:**

. Gβ_1_-L95P exhibited reduced protein expression compared to WT. Even after RNA adjustments to restore comparable membrane localization, the mutant failed to effectively activate GIRK2 and GIRK1/2. Structural analysis revealed that L95 was not consistent in the Gβγ–effector interface. Thermodynamic calculations suggested that the mutation primarily destabilized Gβ_1_ and Gβ_1_–effector complex.

**Conclusion:**

Gβ_1_-L95P leads to both reduced protein expression and impaired function in the GIRK–Gβγ interaction system. The later effect can be attributed to the changes associated with protein misfolding.

## Introduction

Heterotrimeric G-proteins (composed of Gα, Gβ, and Gγ subunits) and G-protein-coupled receptors (GPCRs) constitute integral components of GPCR signaling (Pierce et al., 2002). G-proteins interact with the effectors, including ion channels, enzymes, and various other regulatory proteins, and they play a significant role in diverse physiological processes such as neurotransmission, cardiovascular regulation, endocrine system, metabolism, immune response, cell growth and differentiation, and other intricate intracellular communication pathways (Oldham and Hamm, 2008). Gβγ subunit dimers, or Gβγ subunits for simplicity, in particular, are involved in the activation of GIRK channels (G-protein-activated inwardly rectifying potassium channel), among other functions (Logothetis et al., 1987). Upon activation, these channels induce hyperpolarization of the plasma membrane in cardiac cells and neurons. This activation is crucial for mediating the inhibitory effects of various neurotransmitters, including GABA (*via* GABA-B receptors), acetylcholine (*via* M2 muscarinic receptors), opioids (*via* opioid receptors), and others. The modulation of GIRK channels by Gβγ subunits represents a key mechanism in the regulation of neuronal excitability and the intricate balance of neurotransmission in the central nervous system (Campos-Rios et al., 2022; Cui et al., 2021; Luo et al., 2022).

Over the past decade, a cohort of patients harboring mutations in the Gβ_1_ subunit was described (Petrovski et al., 2016; Bassan et al., 2018). This group, identified with early-onset epilepsy, has been termed GNB1 encephalopathy (GNB1-E). Notable characteristics of these patients encompass developmental delay (DD) or intellectual disability (ID), frequently observed infantile hypotonia progressing to hypertonia, and structural brain abnormalities, accompanied by a spectrum of seizure types such as tonic, absence, myoclonic, generalized tonic-clonic, and focal seizures, along with epileptic spasms. Importantly, *GNB1* has emerged as a potential candidate gene associated with West syndrome, and several individuals with *GNB1*-E have had West syndrome or infantile spasms (Da Silva et al., 2021; Endo et al., 2020; Hemati et al., 2018a; Jones et al., 2019; Schultz-Rogers et al., 2020; Revah-Politi et al., 2020).

GNB1-E is an autosomal dominant rare genetic disorder, with approximately 70 documented cases worldwide. The reported mutations associated with GNB1-E consist of 36 variants affecting 25 residues (Revah-Politi et al., 2020; Lansdon and Saunders, 2021). This suggests the existence of mutational hotspots, where certain amino acid positions are particularly susceptible to genetic alterations associated with GNB1-E. The most prevalent mutation, I80T, has been identified in at least 17 patients (Endo et al., 2020; Tsuji et al., 2023; Hemati et al., 2018b). Our prior investigations extensively examined the I80T, I80N, and K78R mutations using the *Xenopus laevis* oocyte heterologous expression system. We found that the K78R mutation exhibits a gain of expression alongside a partial loss of function when interacting with GIRK1/2, whereas I80T and I80N mutations are characterized by a partial loss of expression with a concurrent loss of function in GIRK2 activation (Reddy et al., 2021).

Moreover, we extended our studies to a mouse model harboring the K78R mutation, demonstrating a recapitulation of developmental delay and epileptic features observed in human cases. Notably, ethosuximide, a potent GIRK inhibitor, was found to suppress spike-wave discharges (SWDs) *in vivo* and restore normal network behavior *in vitro*, highlighting potential therapeutic avenues for managing GNB1-E-associated symptoms (Colombo et al., 2023; Shalomov et al., 2025).

In light of our previous findings, we continued to study the impact of GNB1 mutants on GIRK channels’ activity and expressions in *Xenopus laevis* oocytes. In the current work, we characterized the GIRK1/2 and GIRK2 structural–functional relationship with L95P mutation of Gβ_1_, Gβ_1_-L95P (c.284T > C in exon 7). As of now, L95P is the second most prevalent mutation (seven documented cases: five female, one male, and one unknown) among the GNB1-E patients after I80T (Endo et al., 2020; Lansdon and Saunders, 2021; Tsuji et al., 2023; Hemati et al., 2018b). L95P is strongly associated with cleft palate compared to other GNB1 mutations. L95P is also associated with developmental delay and seizures (Petrovski et al., 2016; Endo et al., 2020; Hemati et al., 2018a). Here, we report that L95P mutation is associated with combined loss of expression and loss of function of GNB1.

## Materials and methods

### *Xenopus laevis* frog maintenance and oocyte collection

The experiments were authorized by and conducted in accordance with the guidelines provided by the Tel Aviv University Institutional Animal Care and Use Committee under permits #01-16-104 and 01-20-083. Female *Xenopus laevis* frogs were cared for and subjected to procedures such as oocyte defolliculation, incubation, and RNA injection, following established protocols (Dascal and Lotan, 1992). The frogs were housed in tanks with dechlorinated water and maintained under a 10-h light/14-h dark cycle at 19°C ± 2°C. Ovary portions were extracted through a small incision in the abdomen under anesthesia (0.2%–0.25% solution of tricaine methanesulphonate). Following suturing, frogs were placed in a separate tank to recover from anesthesia before being transferred to a postoperative animals’ tank. The frogs exhibited no signs of postoperative distress and were allowed a recovery period of at least 3 months. After three to four surgeries, anaesthetized frogs were humanely euthanized through decapitation and double pithing.

Oocytes underwent defolliculation using collagenase (type 1A, Sigma) in a Ca-free ND96 solution (see below). Following a 2–4 h shaking incubation at room temperature, the oocytes were washed and transferred to a Petri dish with fresh ND96 solution and placed in an incubator at 20°C overnight. The subsequent day, visually healthy oocytes were sorted into a fresh dish and maintained in the incubator using NDE solution (ND96 supplemented with 2.5 mM pyruvate and 50 μg/mL gentamicin) at 20°C until RNA injection or further utilization, with daily solution changes. RNA injection followed established procedures (Rubinstein et al., 2009), involving the injection of 50 nL of RNA into healthy oocytes, which were then incubated for 2–4 days in NDE solution. The standard ND96 solution, with a pH of 7.6–7.8, comprised 96 mM NaCl, 2 mM KCl, 1 mM MgCl_2_, 1 mM CaCl_2_, and 5 mM HEPES, with NaOH titration. CaCl_2_ was excluded in Ca^2+^-free ND96.

### DNA constructs and RNA

The DNA constructs utilized for experiments in *Xenopus* oocytes were inserted into high-expression oocyte vectors pGEM-HE or pGEM-HJ, following previously established methods (Berlin et al., 2011; Rishal et al., 2005). Most DNA constructs were reported earlier, including bovine Gβ_1_, bovine Gγ_2_, rat GIRK1, mouse GIRK2, YFP-GIRK1 (rat), and mouse GIRK2-YFP (Yakubovich et al., 2015; Tabak et al., 2019). The Gβ_1_L95P point mutation was introduced to bovine Gβ_1_ through PCR-site directed mutagenesis using standard procedures with the PWO master PCR kit (Roche # 03789403001). DNA preparation employed the Wizard^®^ Plus SV Miniprep kit (Promega # A1460). RNA synthesis followed established protocols (Rishal et al., 2003), with the amounts of injected RNA specified in the text and figure legends.

### Giant membrane patches (GMPs)

Giant membrane patches from oocyte membranes were prepared and visualized following established procedures (Singer-Lahat et al., 2000; Kahanovitch et al., 2014). Oocytes were manually devitellinized using fine forceps in a hypertonic solution (containing in mM: NaCl 56, KCl 150, MgCl_2_ 4, and HEPES 10; pH 7.6). The devitellinized oocytes were placed on a Thermanox™ coverslip (Nunc, Roskilde, Denmark) submerged in a Ca^2+^-free ND96 solution, with their animal pole facing the coverslip, for 10–20 min. Suctioning with a Pasteur pipette was then performed, resulting in a giant plasma membrane patch attached to the coverslip, with the cytosolic face oriented toward the external medium. The coverslip was thoroughly rinsed with fresh ND96 solution and fixed using 4% formaldehyde for 30 min. Fixed giant plasma membrane patches underwent immunostaining in 5% milk in phosphate buffer solution (PBS). Nonspecific binding was blocked with donkey IgG at a 1:200 ratio (Jackson ImmunoResearch, West Grove, PA, United States). Rabbit anti-Gβ_1_ antibody (Abcam, ab 137635) was applied at a 1:300 dilution for 45 min at 37°C. Subsequently, a DyLight 650-labeled secondary antibody (Goat Anti-rabbit IgG, 1:200; Abcam, ab 96886) was applied for 30 min at 37°C, followed by PBS washing and mounting on a slide for visualization. Immunostained slides were stored at 4°C for no longer than a week.

### Confocal imaging

Confocal imaging was conducted using Zeiss LSM 710 or Leica TCS SP8 confocal microscopes equipped with a ×20 objective. For whole oocytes, the image was focused on the animal (dark) hemisphere at the equator. YFP was excited with the 514 nm line of the argon laser, and emission was collected at 535–546 nm. Fiji, an ImageJ-based software (https://imagej.net/ij/), was employed for image analysis. Fluorescent signals were averaged from three regions of interest (ROIs) at the plasma membrane (PM) and three similar ROIs from the coverslip outside the oocyte’s image. The average background signal was subtracted from the average PM signal in each oocyte, followed by subtracting the average net signal from the membrane of uninjected (naïve) oocytes.

For confocal imaging of proteins in giant plasma membrane patches (GMPs), DyLight 650 was excited using a 633 nm laser, and emission was collected at 663–673 nm. Images were centered on the edges of the membrane patches to observe and subtract background fluorescence from the coverslip. Fiji software (https://imagej.net/software/fiji/) was used for image analysis, selecting two ROIs: one covering most of the membrane patch within the field of view and another comprising background fluorescence, which was subtracted from the signal obtained from the patch. The average signal from giant plasma membrane patches of native oocytes’ membranes in the same experiment, immunostained using the same protocol, was subtracted from all groups.

### Two electrode voltage clamp (TEVC)

All experiments were conducted within a temperature range of 20°C–22°C, following procedures outlined in a prior work (Rubinstein et al., 2009). Currents were recorded at −80 mV, filtered at 20 Hz, and sampled at rates of 5 or 10 kHz. Whole-cell GIRK currents in oocytes were assessed using two-electrode voltage clamp (TEVC) with GeneClamp 500 (Molecular Devices, Sunnyvale, CA, United States) utilizing agarose cushion electrodes filled with 3M KCl, featuring resistances of 0.1–0.8 MΩ for the current electrode and 0.2–1.5 MΩ for the voltage electrode.

For measuring GIRK currents through direct activation by Gβγ, oocytes were injected with RNAs of GIRK1 and GIRK2 (0.05 ng) or GIRK2 (2 ng) or YFP-GIRK1 and GIRK2 (0.5 ng) or GIRK2-YFP (2 ng), along with the specified amounts of Gβ_1_ RNA. The Gγ_2_ RNA amount was maintained at 1/5 of Gβ to achieve roughly equal molar ratios of Gβ and Gγ RNAs. GIRK channel currents were measured in ND96 (2 mM K or LK) solution or high-K^+^ solution (HK24), featuring the following composition in mM: 24 KCl, 72 NaCl, 1 CaCl_2_, 1 MgCl_2_, and 5 HEPES. The pH of all solutions ranged from 7.4 to 7.6. The recording protocol is shown in Supplementary Figure S1.

### Quantification and statistical analysis

Statistical analyses were carried out using GraphPad Prism version 10 for Windows (GraphPad Software, La Jolla, California United States). Two-group comparisons employed the t-test when the data passed both the Shapiro–Wilk normality test and the equal variance test; otherwise, the Mann–Whitney rank sum test was utilized. Multiple group comparisons were conducted using one-way ANOVA (ANOVA on ranks when data did not follow a normal distribution). Tukey’s test was applied for normally distributed data, whereas the Kruskal–Wallis test was used otherwise.

The data in the graphs are presented as mean ± SEM. Statistical differences are indicated as follows: asterisks (∗) denote comparisons between channels with WT Gβ and mutant groups; the octothorpe sign (#) indicates comparisons with the channel alone (no Gβγ) group and Gβγ groups. Significance levels are represented as follows: ∗ or #, p < 0.05; ∗∗ or ##, p < 0.01; ∗∗∗ or ###, p < 0.001; ∗∗∗∗ or ####, p < 0.0001.

### Structural analysis of Gβγ–effector interaction

#### Interface and thermodynamic analysis

PDB files were analyzed in PRODIGY (Xue et al., 2016), and lists of amino acids located in the interface between two protein molecules (i.e., Gβγ and channel or other effector) were created. Interface amino acids data are defined by the PRODIGY server as residues in two protein molecules at 5.5 Å and below distance from each other. In order to predict a possible role of L95P mutation in the stability of Gβ subunit and its influence on protein–protein interactions in Gβγ–effector complexes, structural models were submitted to the MCSM server (Pires et al., 2014). The change in protein stability or in the affinity of the protein–protein interaction was defined as significant in case ΔΔG was larger than 1 kcal/mol, which corresponds to ∼5 fold change in dissociation constant at 25°C (Berg et al., 2012) (∼0.6 kcal/mol is considered the noise threshold (Kessel and Ben-Tal, 2018)).

### Gβγ docking procedure

For docking, we utilized the Gβγ structure from the crystal structure of GIRK2/Gβγ complex [4KFM (Whorton and MacKinnon, 2013)], separated from the channel, and a tentative structure of GIRK1/2 heterotetramer described by Gazgalis et al. generated based on homology modeling (Gazgalis et al., 2022). For Gβγ docking, we utilized the rigid body docking algorithm implemented at the ClusPro 2.0 server (Kozakov et al., 2017). No space limitations were imposed during the docking procedure. As a result of the docking procedure, 120 clusters of possible GIRK1/2–Gβγ models were generated and segregated according to balanced, hydrophobic favorite, electrostatic favorite, and van der Waals favorite energy scores. The corresponding centroid models of all clusters were further analyzed utilizing a method similar to that described by Mahajan et al. (2013). In particular, we generated a GIRK1/2 membrane-embedded structure utilizing OPMM (Lomize et al., 2012) and aligned each of the docking models with this structure. Consequently, all models in which any Gβγ atom was located above the lower membrane leaflet and all models in which the C-terminal α-carbon of Gγ was more than 30Å from the lower membrane leaflet were excluded from further analysis. These criteria assured that Gβγ structures implemented for further analysis would be sufficiently close to the plasma membrane, as expected from geranylgeranyl modification of Gγ C-terminus (Hibino et al., 2010).

## Results

### L95P mutation leads to a partial loss of expression of Gβ_1_ protein

**FIGURE 1:**
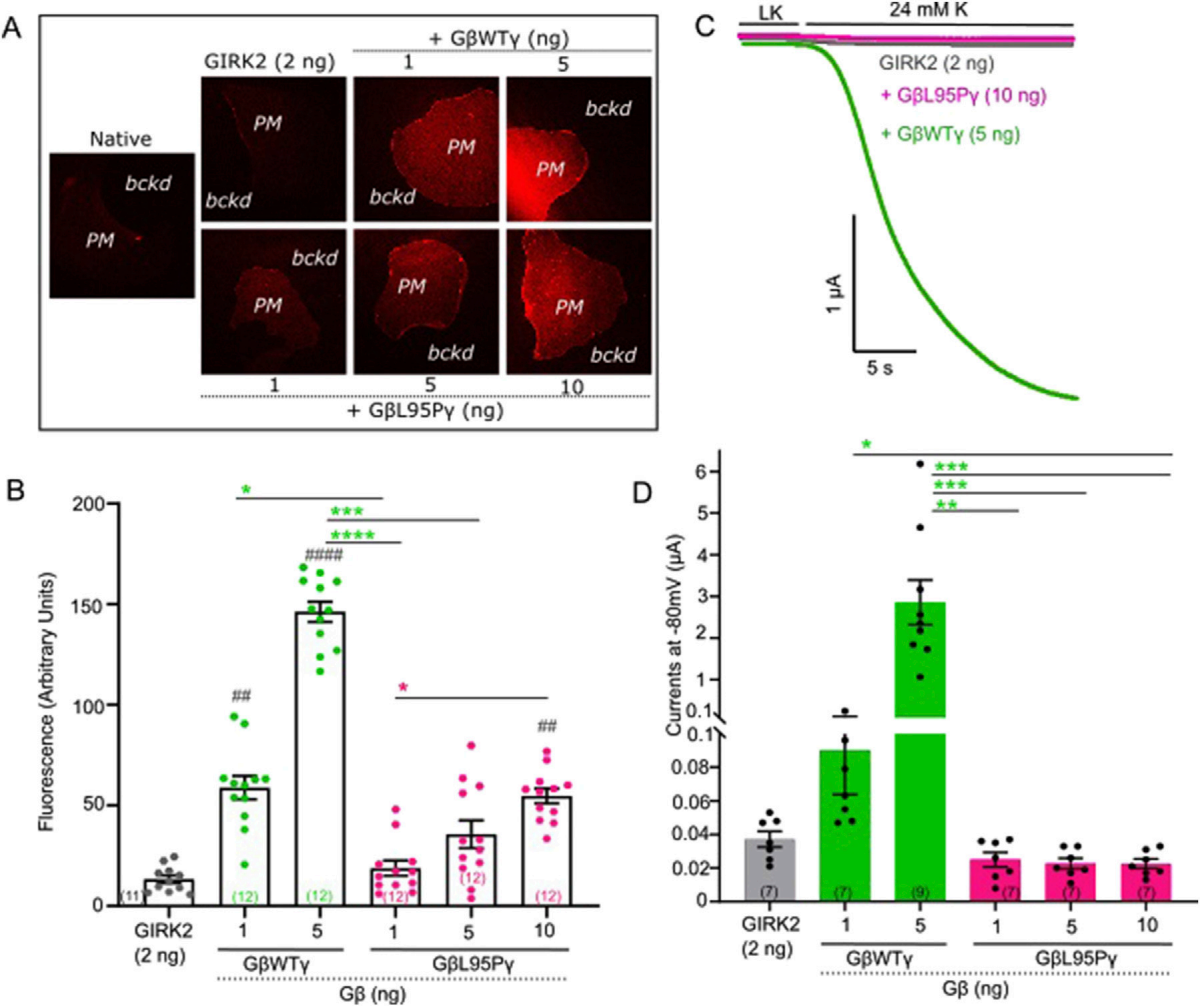
**(A)** Representative images of membrane patches at Gβ RNA doses 1 and 5 ng for GβWT and 1, 5, and 10 ng for GβL95P in the presence of GIRK2 channels. Plasma membrane patches were stained with an antibody against Gβ_1_. Membranes are seen as brighter-colored areas, and the background is black. (*PM*, plasma membrane; *bckd*, background). Number of frog donors (N) = 1. **(B)** Surface expression of GβWT and GβL95P mutant in the presence of GIRK2, measured in GMP. Data shown are the mean fluorescence intensity produced by the expressed Gβ, after subtraction of the average signal observed in channel-alone oocytes. (GMP, giant membrane patches). Statistical analysis: one-way ANOVA ####p < 0.0001, ###p < 0.001, ##p < 0.01, and #p < 0.05 relative to channel-only group and ****p < 0.0001, ***p < 0.001, **p < 0.01, and *p < 0.05 relative to GβWT. **(C)** Representative current traces of GIRK2 channels. **(D)** Summary of GIRK2 activation by GβWT and GβL95P. Statistical analysis: one-way ANOVA ####p < 0.0001, ###p < 0.001, ##p < 0.01, and #p < 0.05 relative to channel-only group and ****p < 0.0001, ***p < 0.001, **p < 0.01, and *p < 0.05 relative to GβWT.

**FIGURE 2:**
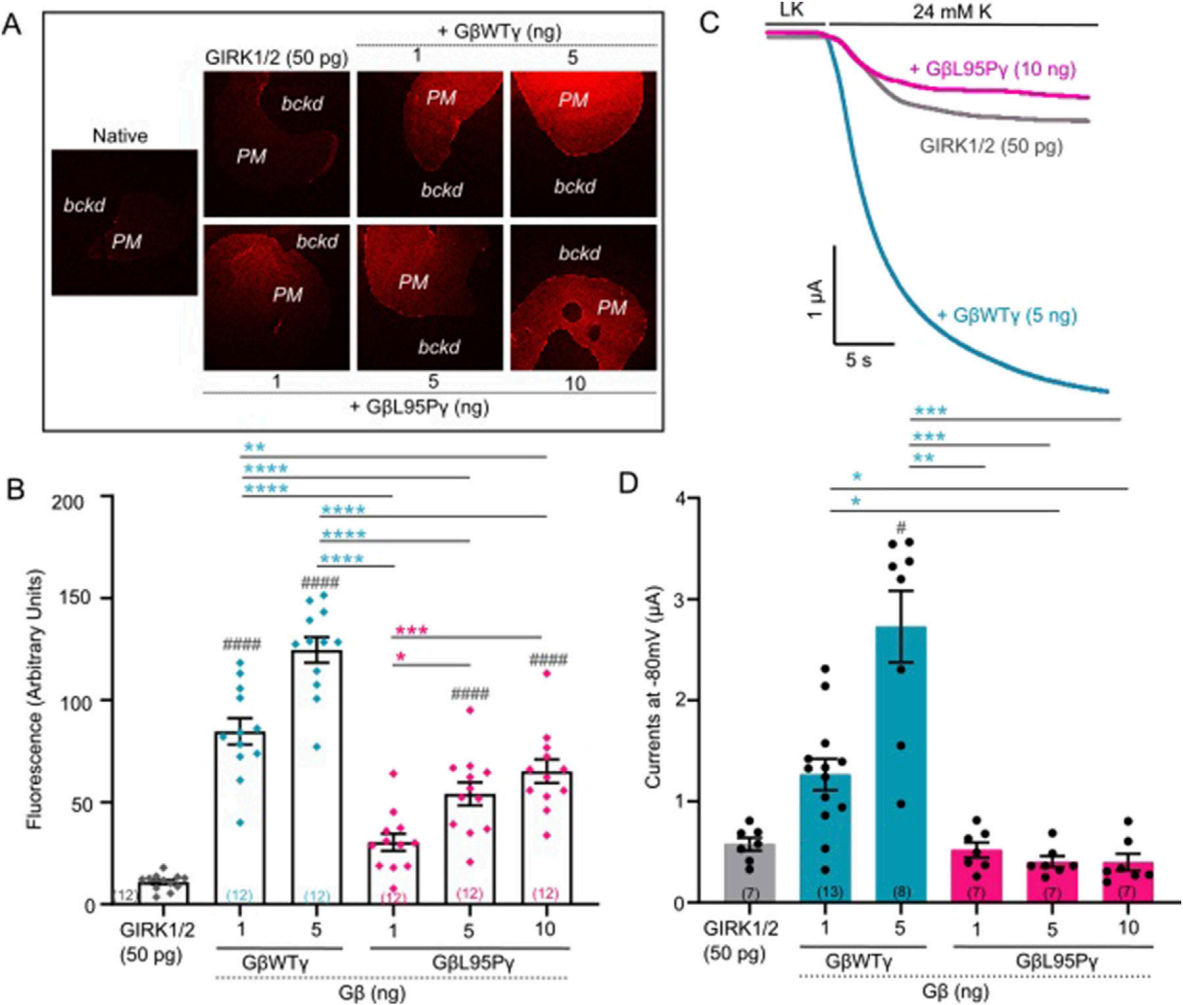
**(A)** Representative images of membrane patches at Gβ RNA doses 1 and 5 ng for GβWT and 1, 5, and 10 ng for GβL95P in the presence of GIRK1/2 channels. Plasma membrane patches were stained with an antibody against Gβ_1_. Membranes are seen as brighter-colored areas, and the background is black. (*PM*, plasma membrane; *bckd*, background). Number of frog donors (N) = 1. **(B)** Surface expression of GβWT and GβL95P mutant in the presence of GIRK1/2, measured in GMP. Data shown are the mean fluorescence intensity produced by the expressed Gβ, after subtraction of the average signal observed in channel alone oocytes (GMP, giant membrane patches). Statistical analysis: one-way ANOVA ####p < 0.0001, ###p < 0.001, ##p < 0.01, and #p < 0.05 relative to channel-only group and ****p < 0.0001, ***p < 0.001, **p < 0.01, and *p < 0.05 relative to GβWT. **(C)** Representative current traces of GIRK1/2 channels. **(D)** Summary of GIRK1/2 activation by GβWT and GβL95P. Statistical analysis: one-way ANOVA ####p < 0.0001, ###p < 0.001, ##p < 0.01, and #p < 0.05 relative to channel-only group and ****p < 0.0001, ***p < 0.001, **p < 0.01, and *p < 0.05 relative to GβWT.

In this study, groups of oocytes were injected with varying amounts of either WT Gβ_1_ or Gβ_1_-L95P mRNA, together with Gγ and channel mRNA (GIRK2 or GIRK1/2). The levels of Gβ expression in the plasma membrane were quantified using the giant membrane patch methodology, which allows to visualize and monitor plasma membrane-attached Gβγ with a Gβ antibody. Concurrently, oocytes from the same RNA injection groups were employed for electrophysiological experiments. Representative pictures obtained from confocal imaging for Gβ_1_-L95P and WT Gβ expressed with GIRK2 and GIRK1/2 channels, respectively, are shown in Figures 1A, 2A. Notably, regardless of which channel was co-expressed with the Gβγ under investigation, the mutant Gβγ consistently exhibited lower expression levels, as illustrated in Figures 1B, 2B. Using higher amounts of injected Gβ_1_-L95P RNA, we could attain levels of expression comparable to the levels of WT Gβ obtained with lower doses of RNA, both in GIRK2 and GIRK1/2 channel-expressing oocytes (Figures 1B, 2B). This result suggests a partial loss of Gβ_1_ protein expression, indicating either reduced synthesis of Gβ_1_-L95P or an increased rate of its degradation, or a combination of both.

### Gβ_1_-L95P fails to activate GIRK channels even when the protein levels are similar to WT Gβ_1_

Having established that expression levels comparable to WT Gβ_1_ (1 ng RNA) can be achieved for Gβ_1_-L95P with a higher dose (10 ng RNA), we proceeded to investigate the ability of Gβ_1_-L95P (coexpressed with WT Gγ) to activate GIRK2 (Figures 1C,D) and GIRK1/2 (Figures 2C,D) channels. Despite achieving sufficient expression, Gβ_1_-L95P failed to activate both GIRK2 and GIRK1/2 channels.

### *GNB1* L95P does not reduce the expression of GIRK channels in the membrane

When levels of expression of GβL95P and GβWT were close to each other, GβL95P failed to activate the channels. As decreased channel expression could yield a similar outcome, our subsequent focus was on investigating the effect of L95P on channel expression in the plasma membrane. We expressed YFP-labeled GIRK channels together with either WT Gβ_1_ or Gβ_1_-L95P. The measurement of fluorescence intensity served as a reporter of channel density.

**FIGURE 3:**
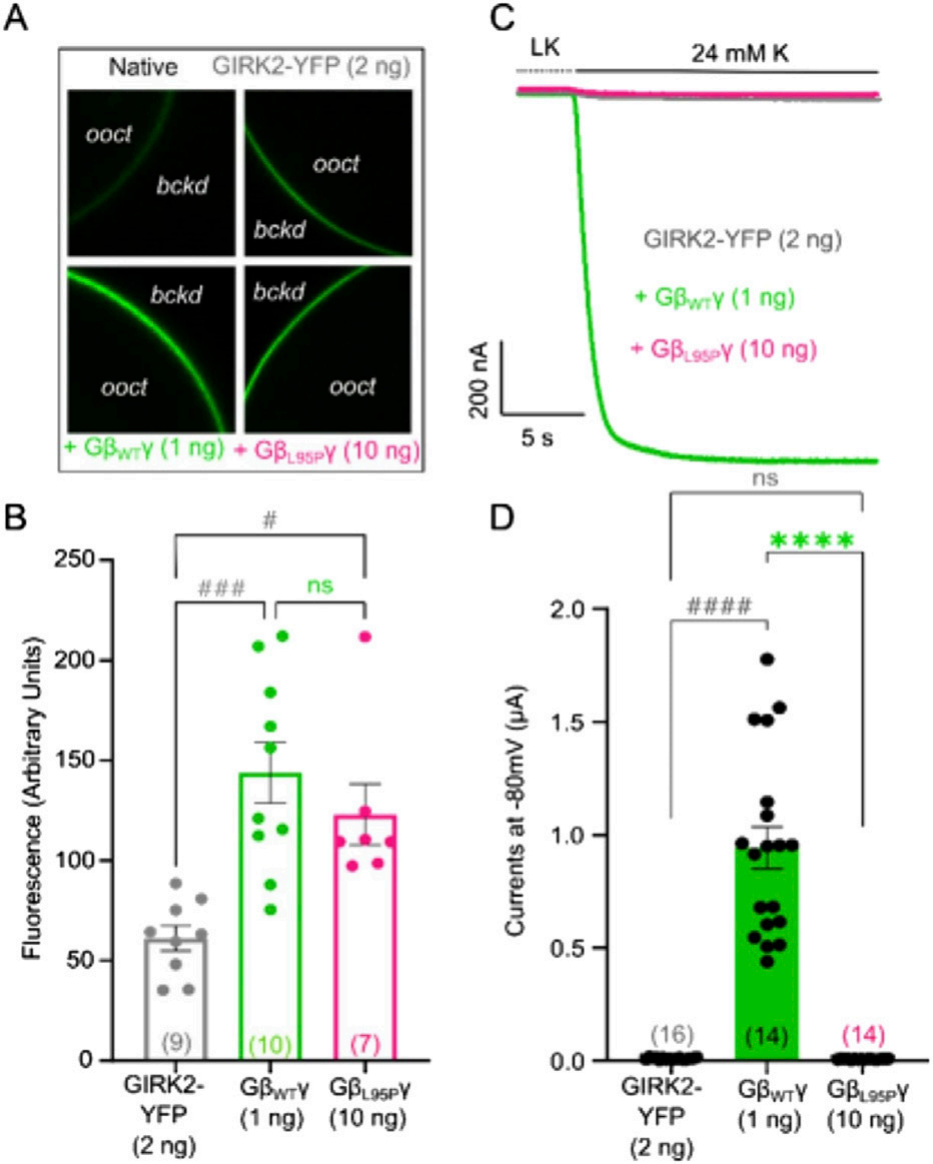
**(A)** Representative confocal images of oocytes expressing GIRK2-YFP channels. **(B)** Surface expression of GIRK2-YFP channels in the presence of GβWT (1 ng) and GβL95P (10 ng) mutant. Data shown are the mean fluorescence intensity produced by the expressed GIRK2, after subtraction of the average signal observed in native oocytes (*ooct*, oocyte; *bckd*, background). **(C)** Representative current traces of GIRK2-YFP channels. **(D)** Summary of GIRK2 activation by GβWT (1 ng) and GβL95P (10 ng). Statistical analysis: one-way ordinary ANOVA (Tukey’s test) or Kruskal–Wallis test ####p < 0.0001, ###p < 0.001, ##p < 0.01, and #p < 0.05 relative to channel-only group and ****p < 0.0001, ***p < 0.001, **p < 0.01, and *p < 0.05 relative to GβWT; ns, not significant. Number of frog donors (N) = 1.

**FIGURE 4:**
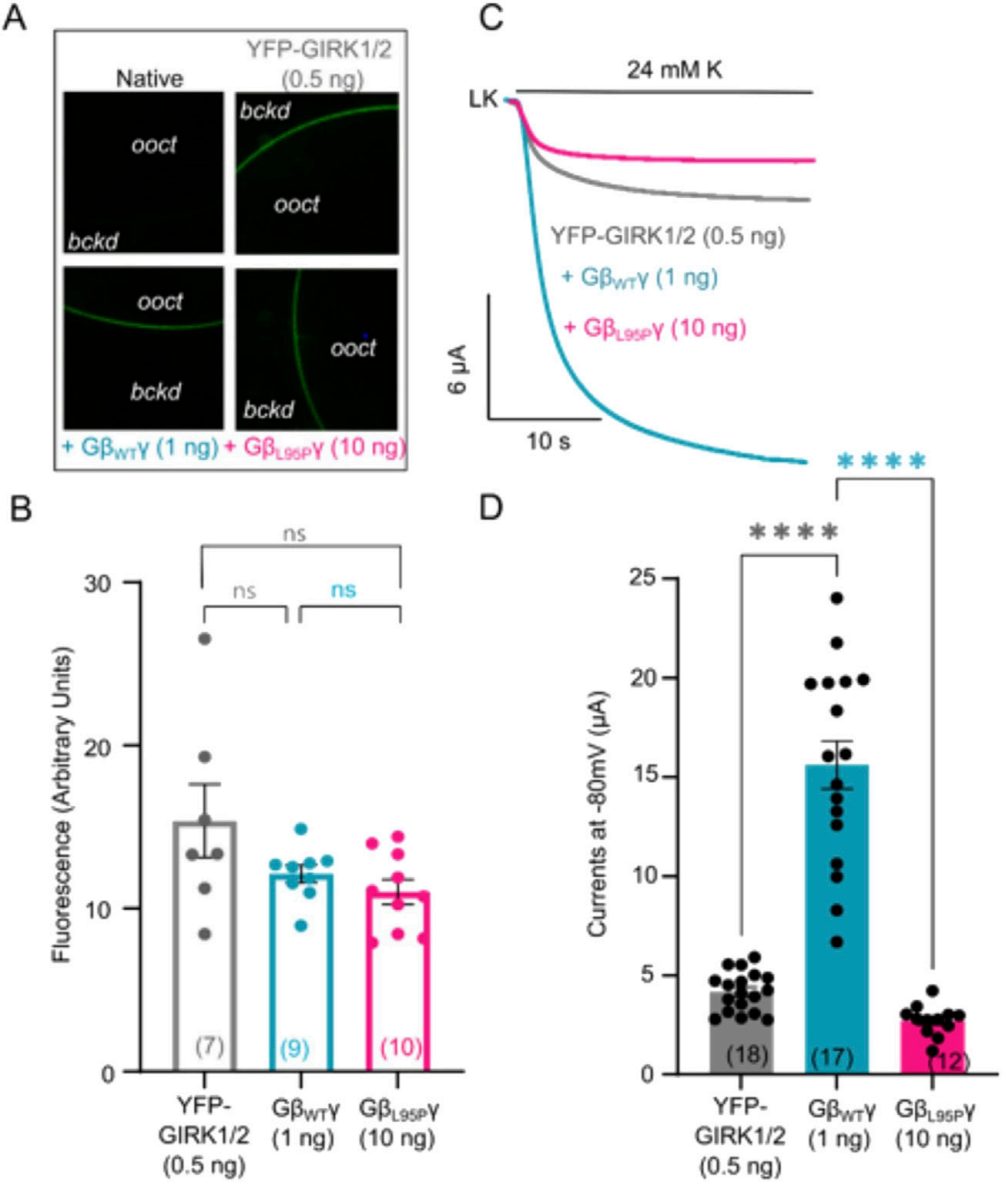
**(A)** Representative confocal images of oocytes expressing YFP-GIRK1/2 channels. **(B)** Surface expression of YFP-GIRK1/2 channels in the presence of GβWT (1 ng) and GβL95P (10 ng) mutant. Data shown are the mean fluorescence intensity produced by the expressed GIRK1/2 (*ooct*, oocyte; *bckd*, background). **(C)** Representative current traces of YFP-GIRK1/2 channels. **(D)** Summary of GIRK1/2 activation by GβWT (1 ng) and GβL95P (10 ng). Statistical analysis: one-way ordinary ANOVA (Tukey’s test) ****p < 0.0001, ***p < 0.001, **p < 0.01, and *p < 0.05; ns, not significant. Number of frog donors (N) = 1.

Our findings revealed a ∼2-fold increase in GIRK2-YFP channel expression in oocytes expressing either mutant or wild-type Gβγ (Figures 3A,B). On the other hand, there was no significant change in YFP-GIRK1/2 expression when compared to channel density without Gβγ co-expression, regardless of whether Gβγ was wild type or L95P (Figures 4A,B). Importantly, the selected RNA doses ensured comparable expression levels for both WT Gβ_1_ and Gβ_1_-L95P.

Simultaneous electrophysiological experiments revealed a lack of channel activation by Gβ_1_-L95P, as demonstrated in both GIRK2 (Figures 3C,D) and GIRK1/2 (Figures 4C,D), when the channel expression in the membrane was unaltered or even increased compared to WT Gβ_1_.

### Structural analysis of L95P mutation

**FIGURE 5:**
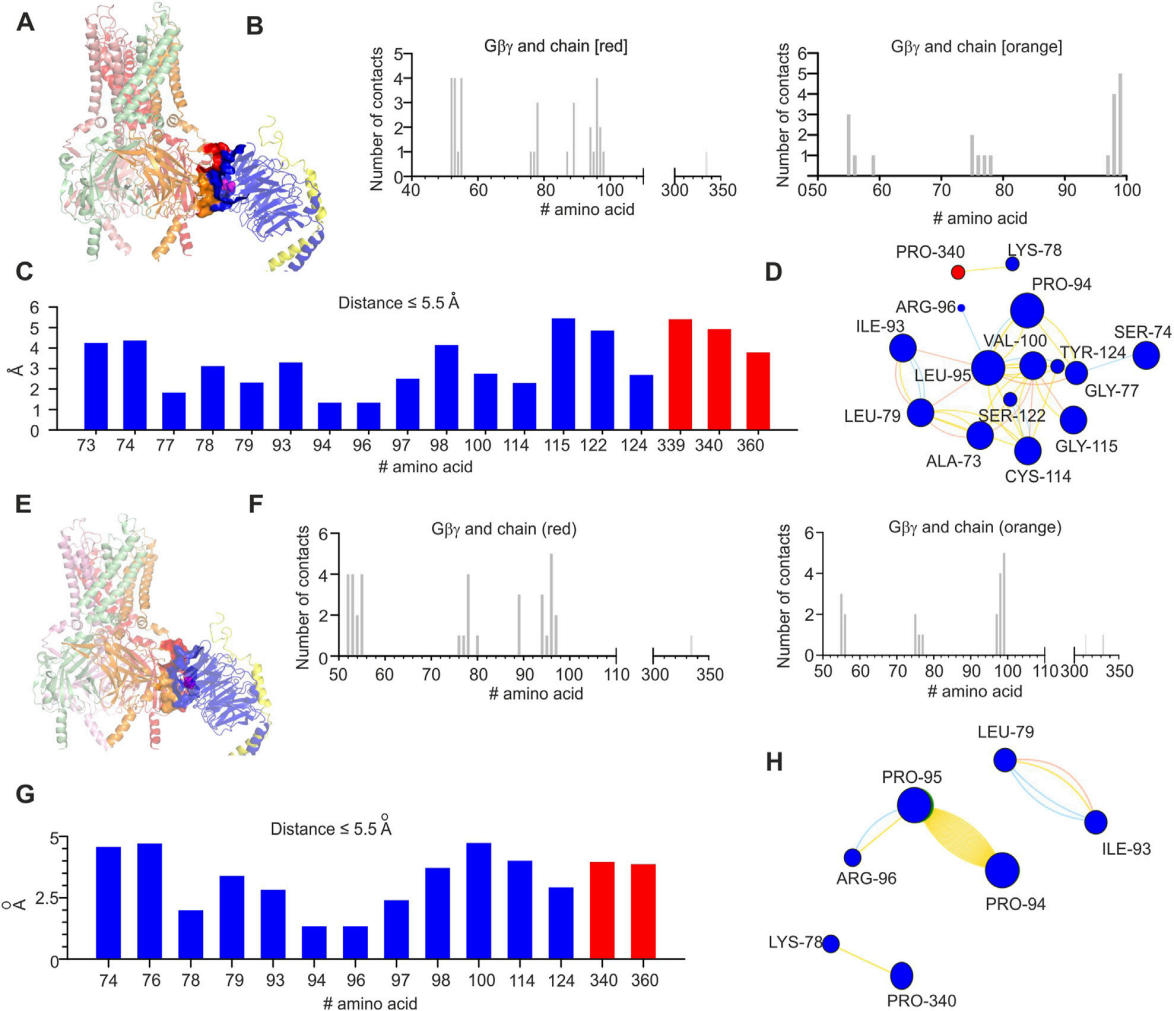
Structural analysis of the impact of L95P mutation on GIRK2-Gβγ complex. **(A)** Crystal structure of GIRK2–Gβγ complex (obtained from 4KFM, three Gβγ molecules were removed); L95 is shown as spheres and colored magenta. **(B)** Interface of GIRK–Gβγ complex, with data generated by the PRODIGY server; selection criterion: all amino acids ≤5.5 Å. The left plot corresponds to the chain colored red in A, and the right plot corresponds to the chain marked orange in **(A)**. Note that L95 is in contact with some amino acids from the chain marked red. **(C)** Amino acids in contact with L95, same criterion as in B, with data obtained from analysis of GIRK–Gβγ wild-type utilizing the INTAA server. Amino acids that belong to Gβ are colored blue, and amino acids that belong to GIRK2 are marked red. **(D)** Analysis of non-covalent interactions of amino acids that are expected to be in contact with L95 (data shown in **C**); network is generated by the RINMAKER server. Specific interactions of L95 are summarized in Supplementary Table S1. **(E)** Predicted coordinates of GIRK2–Gβ_L95P_γ complex, with data generated by the MutationExplorer server utilizing fast Rosetta fixbb function; P95 is shown as spheres and colored magenta. **(F)** Interface of GIRK–Gβ_L95P_γ complex, with data generated by the PRODIGY server; selection criterion: all amino acids ≤5.5 Å. The left plot corresponds to the chain colored red in E, and the right plot corresponds to the chain colored orange in **(E)**. Note that P95 is in contact with the chain marked red. **(G)** Amino acids in contact with P95, same criterion as in F, with data obtained from analysis of GIRK–Gβ_L95P_γ utilizing the INTAA server. Amino acids that belong to Gβ are colored blue, and amino acids that belong to GIRK2 are colored red. **(H)** Analysis of non-covalent interactions of amino acids that are expected to be in contact with P95 (data shown in G); network is generated by the RINMAKER server. Specific interactions of P95 are summarized in Supplementary Table S1.

Initially, we analyzed the existing crystal structure of GIRK2–Gβγ complex (protein data bank accession number 4kfm). Based on coordinates from 4kfm, we generated the list of amino acids that are expected to be in the interface between GIRK2 and Gβγ utilizing the PRODIGY server (in Figure 5A, the amino acids in the interface between GIRK2 and Gβγ are shown as surfaces and L95 is shown as spheres). Notably, in the case of 4kfm, L95 is in the GIRK2–Gβγ interface (Figure 5B). Subsequently, we utilized the same criterion as in the PRODIGY algorithm (distance equal or less than 5.5 Å) in order to generate a contact list of possible contacts of L95, utilizing the INTAA server (Figure 5C). This assumption seems to be reasonable in light of the length of a non-covalent interaction (Kessel and Ben-Tal, 2018). Based on this list, we estimated the possible network of amino acids and non-covalent interactions of L95 utilizing the RINMAKER server (Figure 5D). A summary of amino acids network interacting with L95 is provided in Supplementary Table S1. The analysis procedure mentioned above was repeated for L95P mutation-containing structure that was generated on the base of 4kfm coordinates utilizing protein MutationExplorer server implementing the fixbb function of Rosetta for energy minimization. A cartoon representation of the expected structure of L95P mutant is shown in Figure 5E. Similar to the above, amino acid network and interface analysis were also conducted for the mutant structure (Figures 5F–H and Supplementary Table S1). It can be seen that there is a considerable decrease in amino acids expected to interact with proline in position 95 than with wild-type leucine. Moreover, we conducted limited analysis of the energetic impact of L95P mutation utilizing the MCSM server. It can be seen from Figure 8C that L95P mutation has an energetically significant impact on 4kfm stability. However, the effect of this mutation on GIRK2–Gβγ interaction affinity is expected to be rather modest and is near the thermal noise threshold.

### Analysis of docked GIRK1/2–Gβγ structures

**FIGURE 6:**
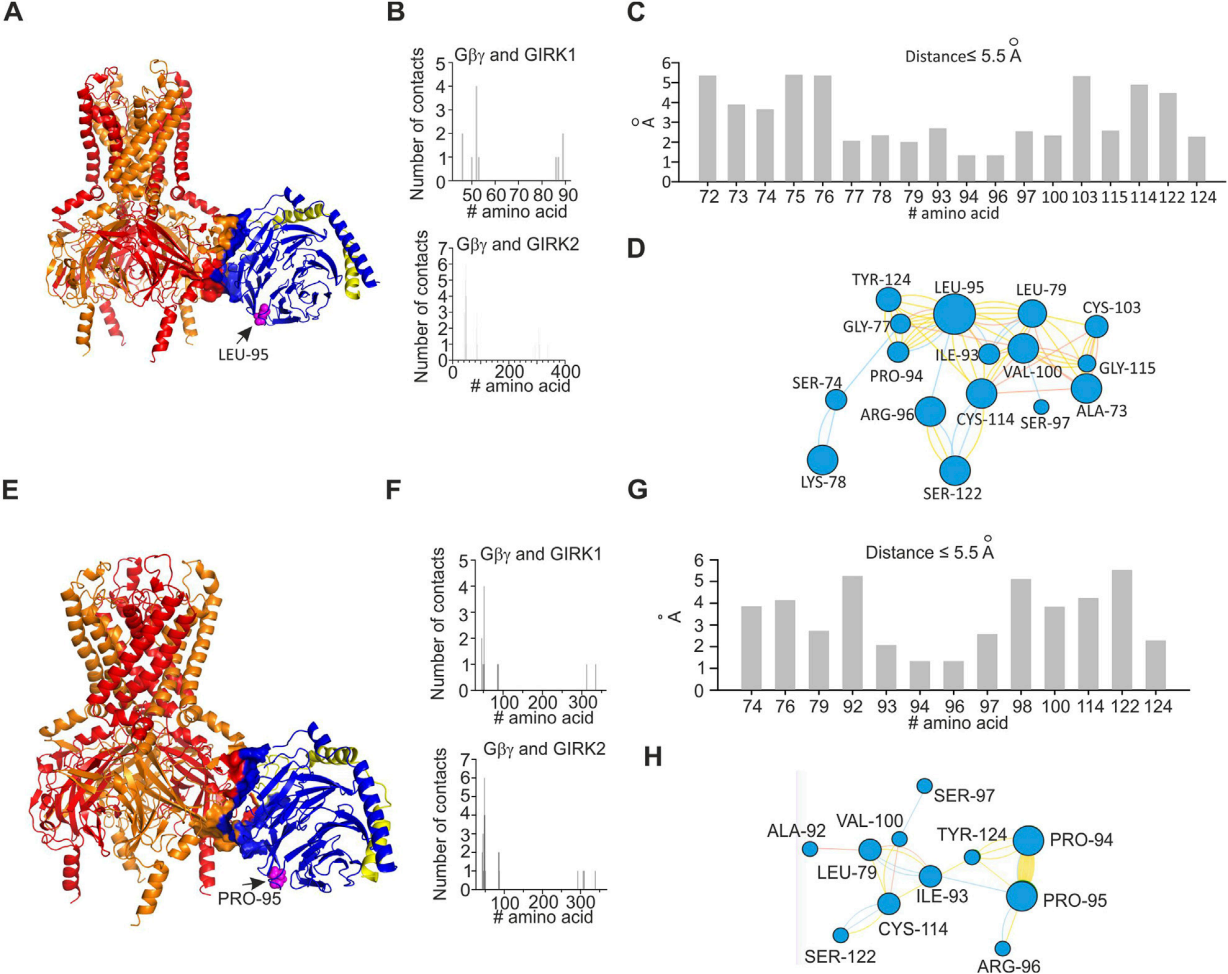
Structural analysis of the impact of L95P mutation on GIRK1/2–Gβγ complex. Analysis was conducted utilizing coordinates of the best scoring GIRK1/2–Gβγ complex. **(A)** Predicted structure of wild-type GIRK1/2–Gβγ complex (single Gβγ molecule is docked); L95 is shown as spheres and colored magenta, GIRK1 subunits are colored red, and GIRK2 subunits are colored orange. **(B)** Interface of GIRK–Gβγ complex, with data generated by the PRODIGY server; selection criterion: all amino acids are at ≤5.5 Å distance. The upper plot corresponds to GIRK1–Gβγ interface. The lower plot corresponds to GIRK2–Gβγ interface. Note that L95 is neither in GIRK1 nor in GIRK2 interface. **(C)** Amino acids in contact with L95, same criterion as in **(B)**, with data obtained from analysis of GIRK1/2–Gβγ wild-type utilizing the INTAA server. As L95 is not part of the channel–Gβγ interface, only amino acids that belong to Gβγ are shown. **(D)** Analysis of non-covalent interactions of amino acids expected to be in contact with L95 (data shown in **C**); network is generated by the RINMAKER server. Specific interactions of L95 are summarized in Supplementary Table S1. **(E)** Predicted structure of GIRK1/2–Gβ_L95P_γ complex, with data generated by the MutationExplorer server utilizing fast Rosetta fixbb function; P95 is shown as spheres and colored magenta. **(F)** Interface of GIRK–Gβ_L95P_γ complex, with data generated by the PRODIGY server; selection criterion: all amino acids are at ≤5.5 Å distance. The upper plot corresponds to GIRK1–Gβ_L95P_γ interface, and the lower plot corresponds to GIRK2–Gβ_L95P_γ interface. Note that P95 is neither in GIRK1 nor in GIRK2 interface. **(G)** Amino acids in contact with P95, same criterion as in **(F)**, with data obtained from analysis of GIRK–Gβ_L95P_γ utilizing the INTAA server. Only amino acids that belong to Gβγ are shown. **(H)** Analysis of non-covalent interactions of amino acids that are expected to be in contact with P95 (data shown in **(G)**); network is generated by the RINMAKER server. Specific interactions of P95 are summarized in Supplementary Table S1.

**FIGURE 7:**
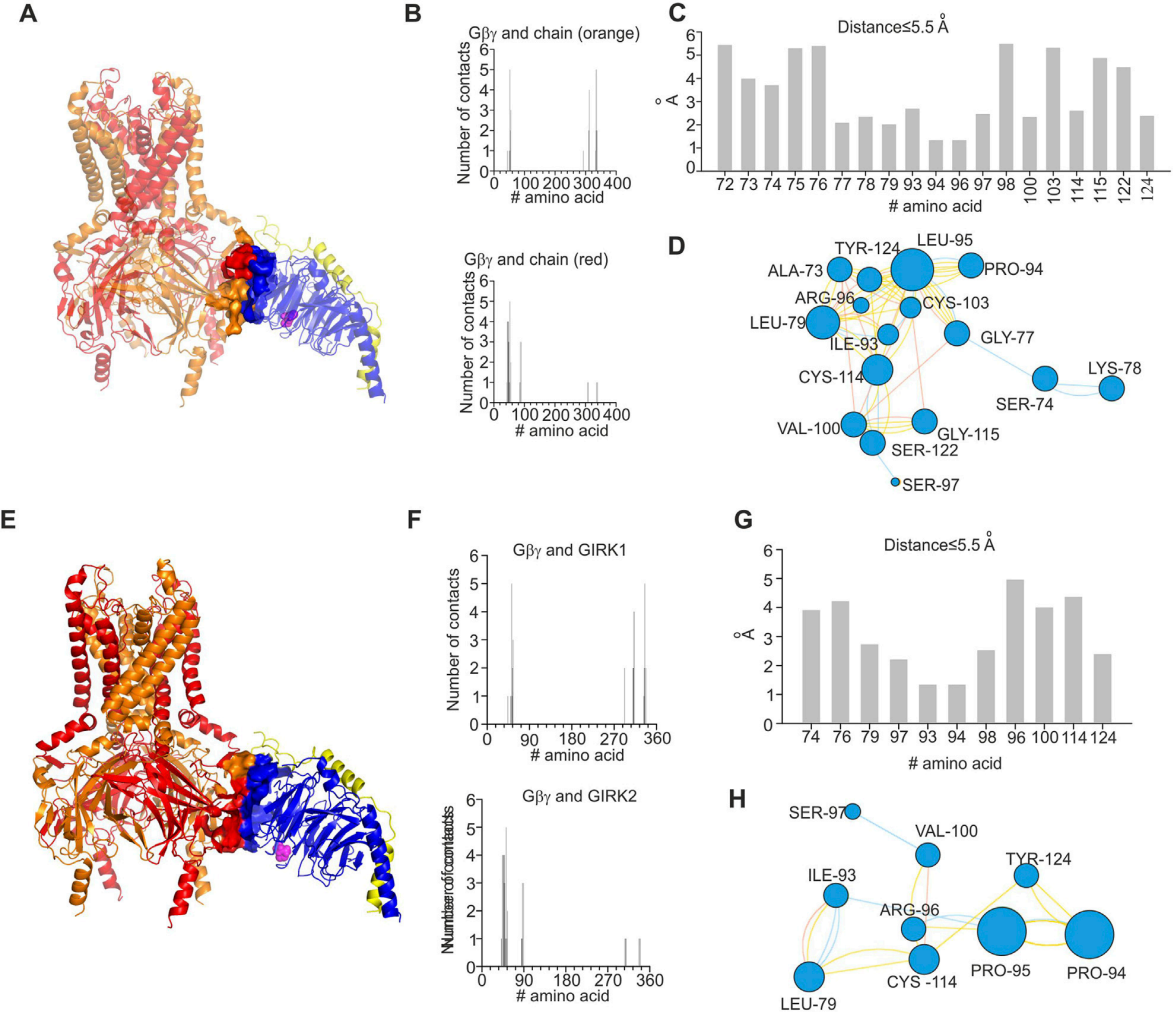
Structural analysis of impact of L95P mutation on GIRK1/2–Gβγ complex. Analysis was conducted utilizing coordinates of the largest cluster of GIRK1/2–Gβγ complex. **(A)** Structure of wild-type GIRK1/2–Gβγ complex (single Gβγ molecule is docked); L95 is shown as spheres and colored magenta, GIRK1 subunits are colored red, and GIRK2 subunits are colored orange. **(B)** Interface of GIRK–Gβγ complex, with data generated by the PRODIGY server; selection criterion: all amino acids ≤5.5 Å. The upper plot corresponds to GIRK1–Gβγ interface. The lower plot corresponds to GIRK2–Gβγ interface. Note that L95 is neither in GIRK1 nor in GIRK2 interface. **(C)** Amino acids in contact with L95, same criterion as in B, with data obtained from analysis of GIRK1/2–Gβγ wild-type utilizing the INTAA server. Only amino acids that belong to Gβγ are shown. **(D)** Analysis of non-covalent interactions of amino acids that are expected to be in contact with L95 (data shown in **(C)**); network is generated by the RINMAKER server. Specific interactions of L95 are summarized in Supplementary Table S1. **(E)** Predicted coordinates of GIRK1/2–Gβ_L95P_γ complex, with data generated by the MutationExplorer server utilizing fast Rosetta fixbb function; P95 is shown as spheres and colored magenta. **(F)** Interface of GIRK–Gβ_L95P_γ complex, with data generated by the PRODIGY server; selection criterion: all amino acids ≤5.5 Å. The upper plot corresponds GIRK1–Gβ_L95P_γ interface, and the lower plot corresponds to GIRK2–Gβ_L95P_γ interface. Note that P95 is neither in GIRK1 nor in GIRK2 interface. **(G)** Amino acids in contact with P95, same criterion as in F, with data obtained from analysis of GIRK–Gβ_L95P_γ utilizing the INTAA server. Only amino acids that belong to Gβγ are shown. **(H)** Analysis of non-covalent interactions of amino acids that are expected to be in contact with P95 (data shown in **(G)**); network is generated by the RINMAKER server. Specific interactions of P95 are summarized in Supplementary Table S1.

In order to investigate energetic and structural consequences of the L95P mutation for a heterotetrameric GIRK channel, we analyzed a predicted model of GIRK1/2–Gβγ complex that was generated utilizing homology modeling and docking methods. We utilized the model of GIRK1/2 published by Gazgalis et al. (2022) and generated a rigid body docking model of GIRK1/2–Gβγ (see Materials and Methods). We implemented selection criteria similar to those described by Mahajan et al. (2013) in order to assure acceptable location of Gβγ relative to the plasma membrane. This procedure rendered 19 model clusters (see Materials and Methods), of which two models, which correspond to centroids of the largest cluster and the cluster with the best energy score, were further analyzed (Figures 6, 7).

**FIGURE 8:**
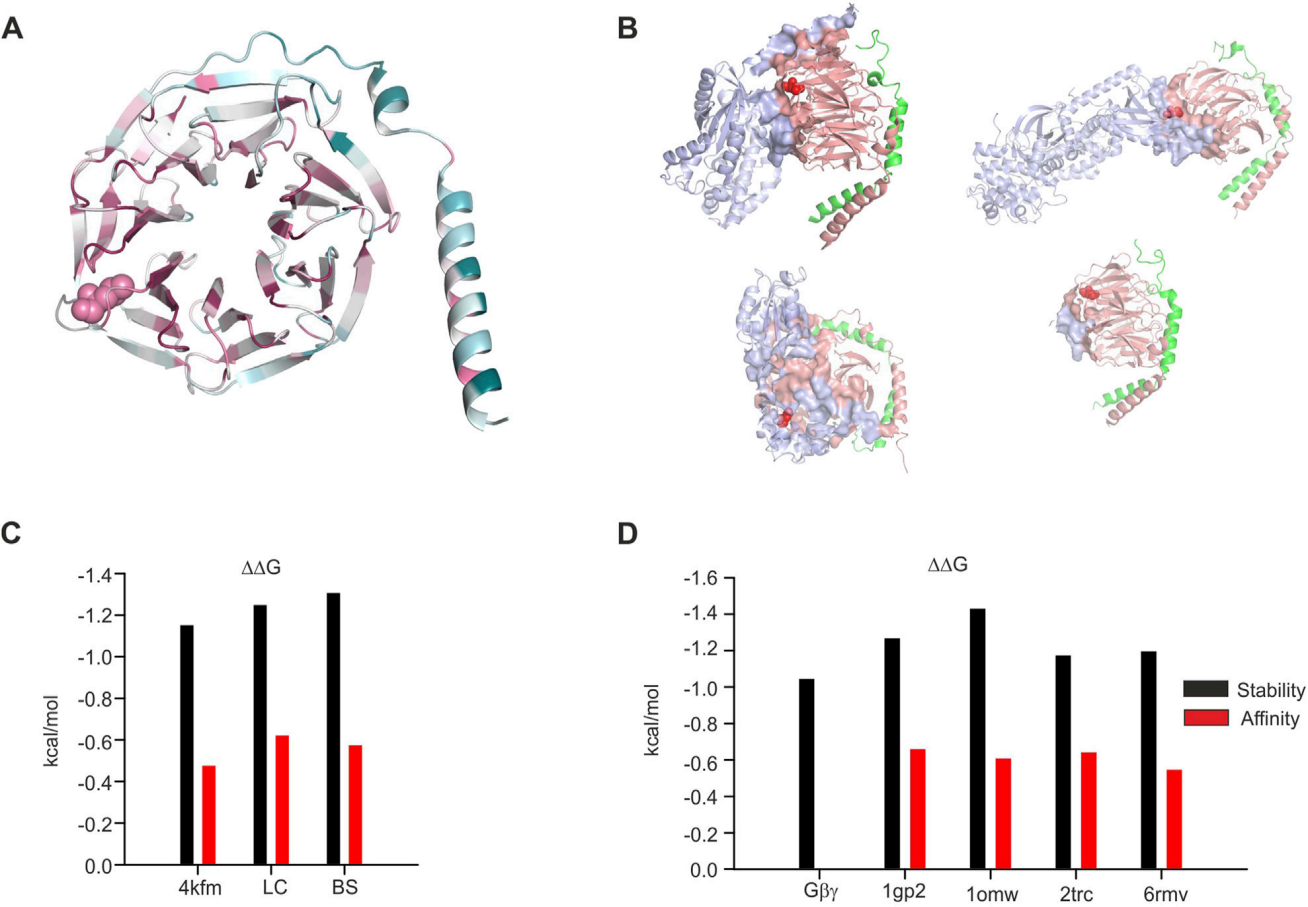
Structural analysis of the impact of L95P mutation on Gβγ−effector complexes. **(A)** Colored scale representation of Gβ_1_ generated in the ConSurf server (Ashkenazy et al., 2016). The scale is 1–9 with more evolutionary conserved amino acids colored by a more darker color. L95 is shown as spheres. **(B)** Gβγ−effector interface analysis. All interfaces are generated by the PRODIGY server. L95 is shown as red spheres; upper left—Gα1GDPGβγ (1gp2), upper right—β-adrenergic kinase–Gβγ complex (1omw), lower left—phosducin–Gβγ complex (2trc), and lower right—TRPM3–Gβγ (6rmv). **(C)** Effect of L95P mutation on protein stability and Gβγ affinity, GIRK crystal structure, and models. **(D)** Effect of L95P mutation on protein stability and Gβγ affinity and other effectors. All data in **(C,D)** were calculated utilizing the MCSM server.

Analysis of the two selected GIRK1/2–Gβγ models, based on the centroid of the largest cluster (lc) and the best energy score (bs), revealed that in both cases, L95 is not part of the GIRK–Gβγ interface (Figures 6, 7). This was consistent across both models, suggesting that the interaction between GIRK1/2 and Gβγ differs from that observed in the GIRK2–Gβγ complex. Additionally, the impact of L95P mutation on amino acids network interacting with that in position 95 in both models is quite similar to that observed for the 4kfm crystal structure. From Supplementary Table S1, it can be seen that at least seven amino acids interact with the wild-type leucine in position 95, whereas 2–4 amino acids remain interacting with proline in the same position in the mutant Gβ. In addition, from the energetic point of view, L95P is expected to be highly destabilizing for both GIRK1/2–Gβγ predicted models, whereas the effect of this mutation on Gβγ–GIRK1/2 interaction affinity is near the thermal noise threshold (Figure 8C).

### Analysis of L95P mutation in other structures

In order to further understand the possible role of L95P mutation in Gβ_1_, we conducted structural and energetic analysis of this mutation in isolated Gβ_1_ and also in other crystal structures that contain this or homologous proteins. In particular, we analyzed the crystal structures of β-adrenergic kinase2 (β-ARK)-Gβγ (1omw) (Thal et al., 2011; Tesmer et al., 2010; Lodowski et al., 2003), Gα_i1_-Gβγ (1gp2) (Wall et al., 1995), and TRPM3- Gβγ (6rmv) (Behrendt et al., 2020) complexes and also of the phosducin-Gβγ complex (2trc) (Gaudet et al., 1996).

Initially, we analyzed the evolutional conservation of amino acids in Gβ_1_ utilizing the ConSurf server (Figure 8A), yielding L95 to be a highly conserved residue (color scale 8 with normalized conservation score of – 0.897). Thereafter, we studied the role of L95 in Gβγ interactions with other effectors mentioned above. We utilized the PRODIGY webserver for the detection of amino acids present in the protein–protein interface. The protein–protein interfaces for these structures are shown in Figure 8B. Out of four examined structures, L95 was found in the protein–protein interface only in one (β-ARK-Gβγ, 1omw).

Consequently, we estimated the possible impact of L95P mutation on protein stability and protein–protein affinity (Figure 8D). It can be seen that in all structures, the predicted energetic cost of the mutation is higher than the RT value at 25 C^o^ (0.6 kcal/mol), and for the β-ARK-Gβγ complex, it is higher than 1.4 kcal/mol. On the other hand, the predicted impact of L95P mutation on affinity change is rather low, ∼0.6 kcal/mol (very close to the RT value, which is considered to be close to thermal noise effect).

## Discussion

In order to study the effect of L95P mutation, we utilized heterologous expression (*X. laevis* oocytes), which enables strict control on both protein level expression and comparison of various possible ligand–effector pairs (Dascal and Lotan, 1992). We demonstrated that the L95P mutation exerts a dual effect by impairing both the expression and function of Gβ_1_ as a GIRK channel activating molecule. Our heterologous expression experiments revealed a clear reduction in expression levels of the Gβ_1_ protein, requiring significantly higher amounts of L95P mRNA than wild-type Gβγ RNA to achieve similar expression in the plasma membrane (Figures 1B, 3B). In addition to the loss of expression, we have shown that L95P mutation also impairs GIRK channel function. Such a combined effect of mutation both on protein expression and protein function has been described for other Gβ_1_ mutations by Reddy et al. (2021). Electrophysiological experiments with GIRK2 and GIRK1/2 channels showed that even when L95P expression levels matched those of wild-type Gβγ, the mutant protein failed to produce equivalent current levels.

The loss of expression effect is also supported by structural analysis as we demonstrated a destabilizing impact of L95P mutation observed in both the 4kfm crystal structure of GIRK2–Gβγ complex and in the predicted models of GIRK1/2–Gβγ. A similar effect was also shown for other Gβγ–effector complexes. This phenomenon, that is, protein misfolding that results from point mutation and subsequent rapid degradation, is described in many known pathologies such as phenylketonuria, cystic fibrosis, short chain acyl-CoA dehydrogenase deficiency disease (Gregersen et al., 2000), and others.

The loss of function effect of L95P mutation is more difficult to explain. In general, ion channel activity (formulated as open probability, for example) is assumed to be a function of maximal open probability and some kind of either hyperbolic or, in more complicated cases, sigmoid function which consists of an efficacy parameter and the concentration of activating ligand (Hille, 2001). In the case of GIRK channels, such a relationship includes channel affinity to Gβγ, maximal open probability, and allosteric coefficients relevant to channel activity changes due to binding of several Gβγ subunits (Touhara et al., 2016; Wang et al., 2016). According to the analysis of various Gβγ effectors, including GIRK2 (from existing crystal structure) and GIRK1/2 (from predicted models), there is no substantial change in Gβγ affinity to the channel induced by L95P mutation.

The aforementioned findings may have several interpretations, and among them is an impact of L95P on the maximal open probability of GIRK1/2 and GIRK2 in a situation similar to partial agonist scheme (Kenakin, 2009). However, this scenario does not agree well with all experimental findings. In a situation of similar affinity of L95P mutant to GIRK channel and lower maximal open probability, the expected effect of L95P expression would be a decrease of channel activity below the level observed before mutant co-expression. It must be taken into consideration that at least GIRK1/2 basal activity is highly (∼90%) Gβγ-depedent, as it was shown by Rishal et al. (2005). Correspondingly, the expression of L95P mutant with equal affinity to wild-type Gβγ and a reduced maximal open probability would lead to the appearance of two channel populations: one corresponding to the maximal open probability of wild-type Gβγ and the other to that of a mutant. Altogether, the above assumption of decreased maximal open probability and no change in Gβγ affinity to GIRK is expected to reduce the basal GIRK1/2 current, which was not observed experimentally.

At this point, we hypothesize that the loss of function effect of L95P mutation may also be associated with the protein misfolding phenomenon, which was described above as a reason for the loss of expression effect of L95P. Misfolded protein molecules frequently fail to interact with their receptors due to aberrant intracellular localization, aggregation, and removal by proofreading systems such as chaperons (Oikonomou and Hendershot, 2020). It must be emphasized that previously described mutations on Gβ (in particular I80T and I80N) were shown to also be loss of expression mutations (Reddy et al., 2021; Colombo et al., 2023), with possible similarity of L95P and I80T/N impact on protein stability.

In summary, the L95P mutation exerts a dual effect on the GIRK–Gβγ system, affecting both protein density and function. Despite L95 not being directly involved in GIRK–Gβγ structural interactions, the study highlights the importance of expression systems such as *X. laevis* oocytes in investigating disease-causing missense mutations. These systems allow precise control and measurement of protein expression and enable electrophysiological studies to assess the functional consequences of such mutations (Hatcher-Solis et al., 2014).

Detailed analysis of point mutations effect on the G-protein subunit–effector interaction conducted in this study and in other studies such as those for Gβγ (Reddy et al., 2021; Colombo et al., 2023) and those for Gα (Knight et al., 2023) can provide important information for further development of treatment strategies of complex medical conditions arising from those mutations (Shalomov et al., 2025).

## Data Availability

The raw data supporting the conclusions of this article will be made available by the authors, without undue reservation.
