# Supplementary figures and images for: Loss of expression and function of Gβγ by GNB1 encephalopathy-associated L95P mutation of the Gβ1 subunit
Source: Front Pharmacol. 2025 May 9;16:1592012. doi: 10.3389/fphar.2025.1592012 (PMC12098346; doi:10.3389/fphar.2025.1592012)

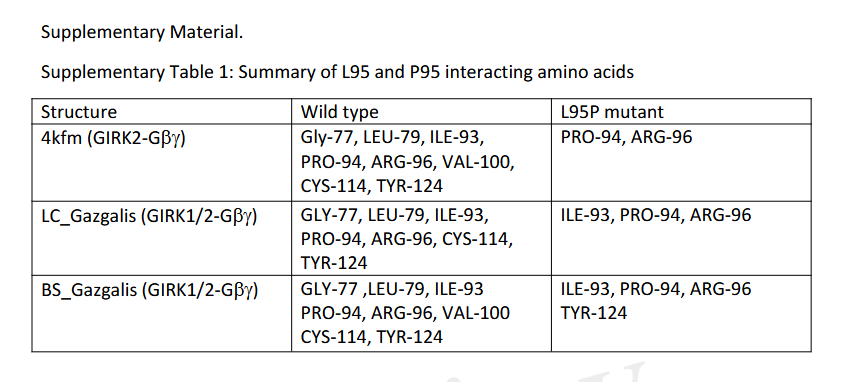

Supplement: Supplementary file 1 [file Table1.docx]
