# Supplementary material for: Loss of expression and function of Gβγ by GNB1 encephalopathy-associated L95P mutation of the Gβ1 subunit
Source: Front Pharmacol. 2025 May 9;16:1592012. doi: 10.3389/fphar.2025.1592012 (PMC12098346; doi:10.3389/fphar.2025.1592012)
Supplement: Supplementary file 2 [file DataSheet1.pdf]

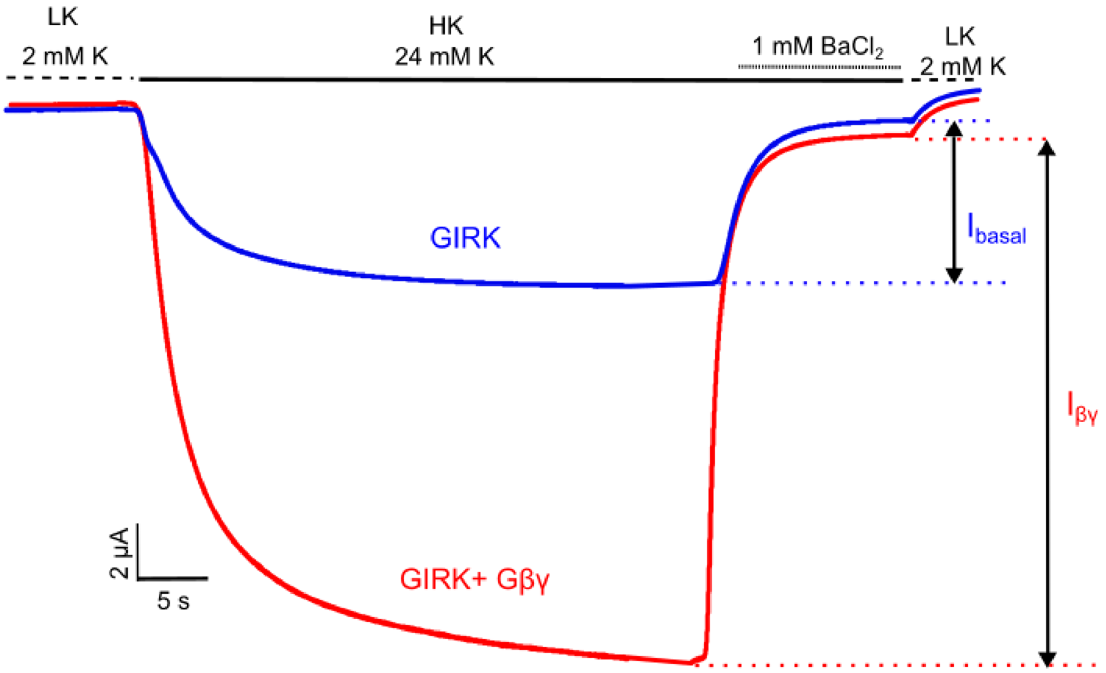

Figure S1. Representative record of GIRK current measurement in oocytes expressing GIRK1/2 (blue trace) or GIRK1/2 and Gβγ (red trace). The oocyte was placed in 2 mM K^+ (LK) solution in the experimental chamber. Following the insertion of two electrodes, voltage clamp was established. Holding potential was -80 mV. Basal current in ND96 was recorded for a short time, and then LK was replaced by a high-K^+ solution, HK24 (with 24 mM K^+) which reversed the flow of K^+, resulting in a large inward current mainly composed of K^+ current via GIRK channels (I_{basal} in GIRK1/2-expressing oocyte, and $I_{\beta\gamma}$ in the GIRK1/2 and Gβγ-expressing oocyte), and a small current via endogenous channels. To separate the latter, the GIRK channels were blocked by perfusing the high-K^+ solution containing 1 mM $BaCl_2$. The Ba^{2+}-blocked current is the net GIRK current. In most cells, at the end of the protocol the solution was exchanged again to the LK solution to verify the stability of the baseline current.
